# Green trees preservation: A sustainable source of valuable mushrooms for Ethiopian local communities

**DOI:** 10.1371/journal.pone.0294633

**Published:** 2023-11-29

**Authors:** Tatek Dejene, Bulti Merga, Pablo Martín-Pinto

**Affiliations:** 1 Sustainable Forest Management Research Institute UVa-INIA, Avenida Madrid, Palencia, Spain; 2 Ethiopian Forestry Development (EFD), Forest Products Innovation Center of Excellency, Addis Ababa, Ethiopia; Universidade Federal de Minas Gerais, BRAZIL

## Abstract

In Ethiopia, *Pinus radiata* and *Pinus patula* are extensively cultivated. Both plantations frequently serve as habitats for edible fungi, providing economic and ecological importance. Our study aims were: (i) to investigate how plantation age and tree species influence the variety of edible fungi and sporocarps production; (ii) to determine edaphic factors contributing to variations in sporocarps composition; and (iii) to establish a relationship between the most influencing edaphic factors and the production of valuable edible mushrooms for both plantation types. Sporocarps were collected weekly from permanent plots (100 m^2^) established in 5-, 14-, and 28-year-old stands of both species in 2020. From each plot, composite soil samples were also collected to determine explanatory edaphic variables for sporocarps production and composition. A total of 24 edible species, comprising 21 saprophytic and three ectomycorrhizal ones were identified. *Agaricus campestroides*, *Morchella* sp., *Suillus luteus*, *Lepista sordida*, and *Tylopilus niger* were found in both plantations. Sporocarp yields showed significant variation, with the highest mean production in 28-year-old stands of both *Pinus* stands. Differences in sporocarps variety were also observed between the two plantations, influenced by factors such as pH, nitrogen, phosphorus, potassium, and cation exchange capacity. *Bovista dermoxantha*, *Coprinellus domesticus*, and *A*. *campestroides* made contributions to the variety. The linear regression models indicated that the abundance of specific fungi was significantly predicted by organic matter. This insight into the nutrient requirements of various fungal species can inform for a better plantation management to produce both wood and non-wood forest products. Additionally, higher sporocarps production in older stands suggests that retaining patches of mature trees after the final cut can enhance fungal habitat, promoting diversity and yield. Thus, implementing this approach could provide supplementary income opportunities from mushroom sales and enhance the economic outputs of plantations, while mature trees could serve as a source of fungal inoculum for new plantations.

## 1. Introduction

Forest fungal communities contain a wide range of species that contribute significantly to the development, functioning, and stability of ecosystems [1,2]. In addition, fungi are part of the livelihood of people living in different parts of the world [3–5]. Fungi have long been collected as valuable non-timber forest products (NTFPs), generating a cash income for market traders, as well as being used for local subsistence in food and as a traditional medicine [3,6,7]. These practices help rural people to reduce their vulnerability to poverty and, in some regions, forest fungi also serve as a seasonal coping food during food shortage periods, mainly in the rainy season when grain is scarce [8]. However, in developing countries, activities related to forest management are focused on maximizing wood products rather than NTFPs, among which forest fungi are the most neglected NTFP resource [9–11].

In Ethiopia, the growing demand for forest products and agricultural land has resulted in widespread deforestation of natural forests [12,13]. To address this issue, fast-growing tree plantations have been established, mainly as community forests, which has led to a significant increase in the number of exotic tree species in recent decades [13–15]. These exotic plantations have the potential to produce high-value timber and NTFPs [16,17], including wild edible fungi [18,19]. Although studies have been conducted to assess the overall fungal diversity and sporocarp production in Ethiopian forest systems, there is still a lack of specific knowledge about the diversity, sporocarp production, and factors influencing the variety of valuable fungal species in short-rotation plantations [2,19].

*Pinus radiata* and *Pinus patula* are fast-growing, exotic, commercial timber-producing tree species. They are cultivated extensively in areas with mean annual rainfall levels of between 700 and 1500 mm, depending on the agroecology and elevation aspects [16,17]. Vast areas of *Pinus* tree plantations have been established, including state-owned forests, and are now the third largest plantation species after *Eucalyptus* and *Cupressus* in terms of overall plantation area in Ethiopia [13,20]. Due to their adaptability to a wide range of ecological conditions and rapid growth rate, *Pinus* species are highly desirable for sawn timber, poles, and posts [16]. In Ethiopia, the rotation period of *Pinus* species in general is between 30 and 40 years [21], with maximum timber production achieved when trees are 26–30 years old [22]. The management of *Pinus* plantations in Ethiopia is based on traditional silvicultural systems, with clear felling followed by replanting the preferred management technique [22,23]. Both the short-rotation period and clear-felling may have a direct impact on ecosystem properties and associated fungal communities that are sensitive to this type of management [24].

In plantation forest system, the variety of fungal species is closely linked to various biotic and abiotic factors, such as stand age [25,26], elevation [27,28], tree productivity [29,30], and soil environment [31,32]. Vegetation factors, such as stand age and site species composition, can affect fungal communities and their productivity directly through host–fungi specificity or indirectly through resource inputs, such as root exudates and litterfall [33,34]. Previous studies have shown that tree species identity is a key factor governing soil microbial communities, particularly mycorrhizal fungi [35,36]. Furthermore, forest stand age can affect fungal community structure and function through changes in the quantity and quality of litter [37].

In plantation forests dominated by a single tree species and characterized by a long rotation, such as temperate or boreal forests, the fungal community is expected to be highly dependent on the type of tree species [38]. In tropical forest systems, where plantations are characterized by a short rotation system and traditional silvicultural practices, such as clear felling and replanting, the impact of tree species and other biotic and abiotic factors on the fungal variety has rarely been studied, particularly in Ethiopian forest systems [39]. However, appropriate management of Ethiopian plantation forests is crucial to provide wood products while maintaining ecosystem integrity to provide a more conducive environment for fungi [39–41]. Therefore, it is important to identify mechanisms that regulate edible fungal communities and their production of sporocarps alongside the growth of stands. This information can be obtained by examining the distribution patterns of fungi and changes in their associations over time, particularly in plantations with short-rotation periods.

The overall aim of this study was to generate knowledge that could aid the management of plantation forests in Ethiopia to produce both wood and non-wood forest products, particularly edible wild mushrooms, based on a green tree retention approach. This practice involves leaving some living trees in harvested areas to retain ecosystem complexity by maintaining some of the structural diversity of the original forest even after harvesting. The retained trees provide habitat and support for mycorrhizal fungi, which are essential for the growth and fruiting of many edible mushrooms [42], such as chanterelles, porcini, and morels [43]. Hence, this approach can benefit both commercial and non-commercial harvesters of edible mushrooms and provide ecological benefits, which could increase the value of Ethiopian plantation forests.

We hypothesized that the production of edible sporocarps in short-rotation *P*. *patula* and *P*. *radiata* plantation forests in Ethiopia would be correlated with stand age in a specific manner, anticipating that like other plantations, species richness and sporocarp production would be highest in the oldest stands. We also anticipated that the variety of edible fungi would differ between the two plantation forests because the variety would be driven mainly by vegetation and site conditions, such as soil. Our specific objectives were: (i) to investigate how plantation age and tree species influence the variety of edible fungi and sporocarp production; (ii) to determine edaphic factors contributing to variations in sporocarp composition; and (iii) to establish a relationship between the most influencing edaphic factor and the production of valuable edible mushrooms for both plantation types.

## 2. Materials and methods

### 2.1. Study areas

The study was conducted in Menagesha Suba and Wondo Genet in the Oromia and Sidama Regional states of Ethiopia, respectively. Menagesha Suba forest is located 30 km from Addis Ababa in the central part of Ethiopia, while Wondo Genet is located 265 km from Addis Ababa in southern Ethiopia. The climate of the study areas is characterized by the Woyna Dega agro-climatic type. The rainfall pattern is bimodal, with low rainfall during spring and the main rainy season during the summer. Comprehensive geographical descriptions of the forests are provided in Table 1. The topography of the study areas is extremely dissected, with alternating ridges and valleys dominating the landscape and soils that are characterized by sandy loams. Soils at higher altitudes are shallow with a rocky substrate. At lower altitudes, where most of the plantations are located, the soil is deep but less gravelly at the Menagesha Suba site [44].

**Table 1:** Geographical descriptions of the study areas.

Study areas	Geographical location	Altitude (m.a.s.l.)	MAP (mm)	MAT (°C)	References
Wondo Genet	7°06′–7°07′ N / 38°37′–38º42′ E	1,600–2,580	1,210	20	[45]
Menagesha Suba	8°56’–9°02’ N / 38°28’–38°36’ E	2,200–3,385	1,100	16	[46]

**Note:** MAP, mean annual precipitation; MAT, mean annual temperature; m.a.s.l., meter above sea level.

Both Menagesha Suba and Wondo Genet have officially protected areas. In Menagesha Suba, 9,248 ha are protected, of which original natural forests account for 2,500 ha and plantation forests of different species account for 1,000 ha. Plantations were established on areas that had already been deforested for cultivation and comprise both indigenous and exotic tree species, including *Juniperus procera*, *Eucalyptus globulus*, *P*. *radiata*, *P*. *patula*, and *Cupressus lusitanica*. By contrast, the original vegetation of the Wondo Genet was destroyed long ago because of logging and clearance for cultivation [16]. Consequently, large plantations of exotic tree species were established and there is currently approximately 100 ha of non-native forests of different tree species growing in the study area. The three predominantly planted species are *C*. *lusitanica*, *Grevillea robusta*, and *P*. *patula* [16,47].

### 2.2. Experimental design and sporocarp sampling

We selected two different *Pinus* plantations (*P*. *radiata* and *P*. *patula*) with 5-year-old, 14-year-old, and 28-year-old stands, hereafter referred to as ‘young’, ‘medium-aged’, and ‘old’ stands, respectively. Sporocarp data were collected using transect methods, as described in previous studies [18,48]. In each stand, three 2 × 50 m (100 m^2^) plots were established at least 200 m apart [49], i.e., nine plots in total, as described in [50,51]. Experimental plots were randomly laid out in each stand to avoid confounding spatial effects inherent to such a plot-based design [52,53], and all experimental plots were analyzed as independent samples [54].

Sporocarps were collected once a week during the main rainy season between July and August 2020 as described in [55,56]. Fresh weight measurements of sporocarps of each species were carried out *in situ*, and abundance data were obtained for each plot. Specimens were photographed in the field and their ecological characteristics were noted to assist and facilitate taxa identification processes. Sample fruit bodies of each species were taken to the laboratory and dried. These specimens were used for microscopic examination as part of the species identification process. Also, the fieldworks in the forests of Wondo Genet and Menagesha Suba were carried out with full authorization granted by Wondo Genet College of Forestry, and Oromia Forest and Wildlife Enterprise respectively.

### 2.3. Sporocarp identification

Taxonomic classification was conducted by examining tissues and spores with an Optika B-350PL microscope in the laboratory. Small samples of dried sporocarp specimens were rehydrated and mounted in 5% KOH for evaluation and identification. Morphological features of fruit bodies were examined using appropriate monographs, including [57–60], to determine the genus and species of the macrofungal specimens. Up-to-date fungal taxa names and authors’ names were obtained from the Mycobank database (http://mycobank.org). Ecological classification of each species was assigned based on fungal traits, as described in [61]. In addition, the edibility of the collected fruiting bodies was assessed following the criteria described in [62]. Taxa described in the literature as both non-edible and edible were classified as non-edible. Taxa described in the literature as having doubtful edibility were classified as non-edible. Only species classified as edible by a large majority of the literature consulted were classified as edible fungi.

### 2.4. Soil sampling and analysis

Soil samples were collected at a depth of 10 cm from all experimental plots. Soil samples were collected from five soil cores within each plot: the center and the four corners and then the selected soil physicochemical parameters were analyzed. The analysis was conducted by Water Works Design and Supervision Enterprises Laboratory Service Sub-process in the Soil Fertility Section at Addis Ababa, Ethiopia. Soil pH and electrical conductivity (EC) in a 1:2.5 (soil: liquid ratio) suspension and the supernatant of the same suspension were measured using a pH meter and an EC meter, respectively [63]. The wet digestion method [64] was used to determine soil carbon content, and total nitrogen was analyzed using Kjeldahl digestion [65], distillation, and titration as described by [66] by oxidizing the organic matter (OM) in concentrated sulfuric acid solution (0.1 N H_2_SO_4_). The available phosphorus of soils was determined using a standard procedure [67]. Soil parameters (i.e., EC, K, Ca, Mg, and cation exchange capacity (CEC)) were extracted using the diethylenetriaminepentaacetic acid extraction method [68], and all these soil characteristics were measured using an atomic absorption spectrophotometer. Comprehensive soil descriptions of each plot established in both types of plantation forest are provided in Table 2.

**Table 2:** Selected edaphic variables of young, medium-aged, and old *Pinus patula* and *Pinus radiata* stands growing in study areas located in Menagesha Suba and Wondo Genet, Ethiopia.

Soil parameter	*Pinus patula*			*Pinus radiata*		
	Young	Medium	Old	Young	Medium	Old
Sand (%)	66.63ª	50.10^b^	52.39^b^	65.63ª	56.97ª	57.49ª
Silt (%)	19.93ª	23.33ª	24.06ª	19.93ª	15.26ª	16.72ª
Clay (%)	14.44ª	19.90ª	21.88ª	14.44^b^	27.78^a^	25.79ª
pH-H_2_O	6.57ª	6.08ª	6.19ª	5.33ª	5.54ª	5.62ª
EC (meq/100 g soil)	0.28ª	0.09^b^	0.07^b^	0.16ª	0.24ª	0.15ª
K (meq/100 g soil)	1.32ª	0.42^b^	0.77^b^	0.44^ab^	0.66ª	0.33^b^
Ca (meq/100 g soil)	22.62ª	13.66ª	16.01ª	22.62ª	14.70ª	17.81ª
Mg (meq/100 g soil)	7.96ª	4.36^b^	6.66^ab^	6.96^a^	5.05^b^	5.87^ab^
CEC (meq/100 g soil)	49.54ª	28.07^b^	42.04ª	41.19^ab^	42.49ª	33.10^b^
OM (%)	5.96^b^	7.27^b^	13.33ª	6.36^b^	8.93ª	10.14ª
N (%)	0.83ª	0.71ª	0.34^b^	0.37ª	0.52ª	0.32ª
P (mg P_2_O_5_/kg soil)	35.70ª	36.99ª	38.56ª	31.04^ab^	33.37^a^	23.78^b^

**Note:** Different lowercase letters indicate significantly different soil parameter mean values among stand ages of the same tree species (*p* < *0*.*05*). CEC, cation exchange capacity; EC, electrical conductivity; OM, organic matter.

### 2.5. Statistical analysis

We used Linear Mixed Effects models (LME, *p* ≤ 0.05, package Nlme) to analyze the effects of stand age and *Pinus* species on the total fungal yield [69], where stand age was defined as a fixed variable and *Pinus* species was added to the model as a random variable. To determine specific significant differences, Tukey’s test was applied to the fixed variable.

Relationships between sporocarp variety and edaphic variables were visualized using non-metric multidimensional scaling (NMDS) based on an abundance species data matrix and scaled soil data. A permutation based nonparametric MANOVA (PerMANOVA) [70] using Euclidean distance was performed to analyze differences in sporocarp communities between stand types. Isolines were also plotted on the NMDS ordinations for OM using the ordisurf function. Correlations between NMDS axes scores with explanatory variables were assessed using the envfit function in R. To assess the influence of edaphic variables on the fungal community, we performed a Mantel test (Bray–Curtis distance) on the total species matrix and scaled environmental parameters. In addition, an analysis of similarity percentages (SIMPER) [71] was performed to identify edible fungi that were most responsible for the observed patterns, and was also used to determine the percentage contribution of macrofungal species to significant dissimilarities between the two forest types [72]. The SIMPER analysis was performed using the sim function of the Vegan package in R [73].

## 3. Results

### 3.1. Number of species and fresh biomass production

**Fig 1:**
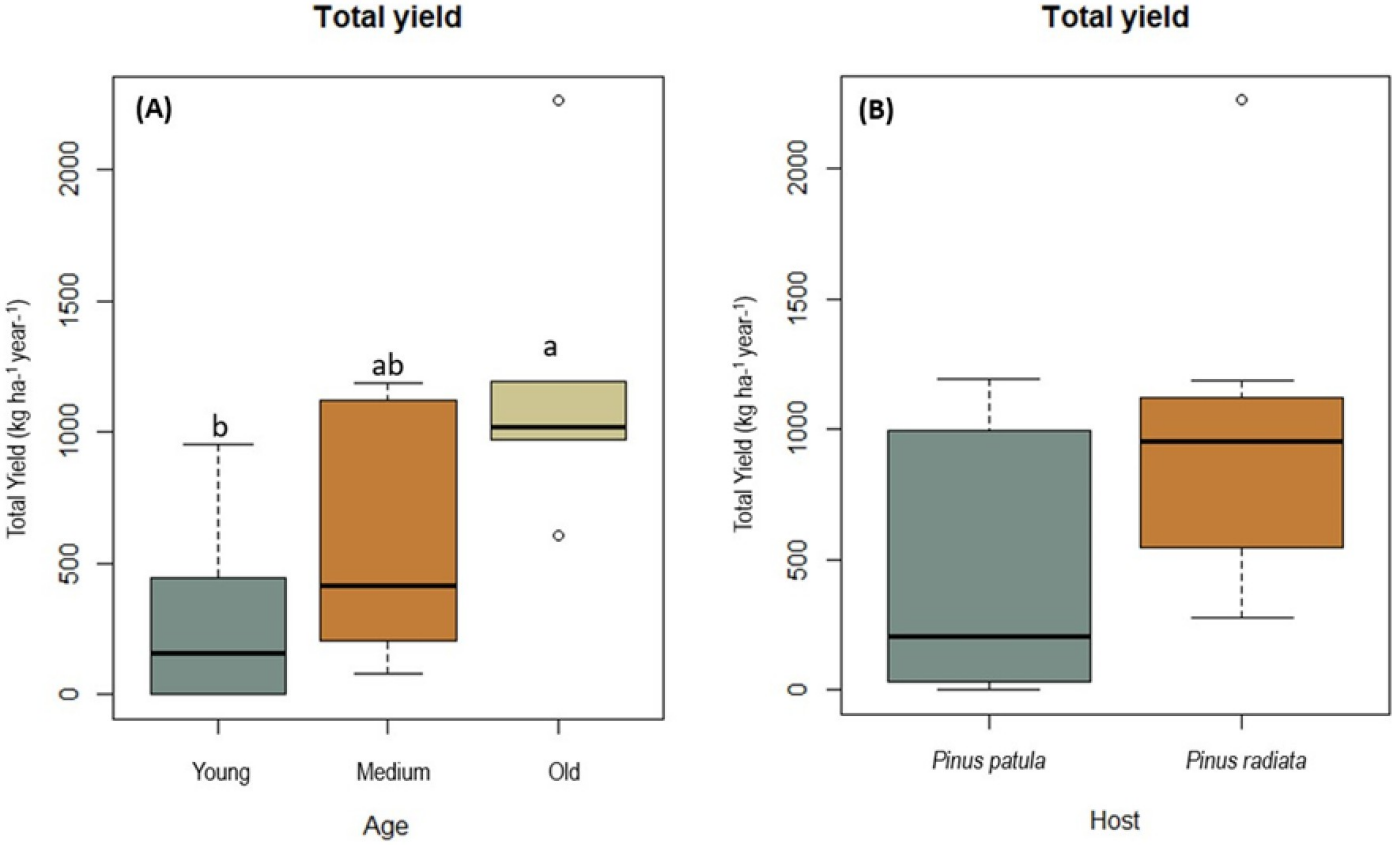
Annual yield of edible sporocarps according to stand age (A) and plantation type (B) collected from study areas located in Menagesha Suba and Wondo Genet, Ethiopia. Box plot data showing the maximum and minimum values. **Note:** *The bar in the box is the standard deviation of the mean*. *Yields with the same letter are not significantly different (p > 0*.*05)*.

In total, 24 edible wild mushrooms were collected from plots established in young, medium-aged, and old stands in *P*. *patula* and *P*. *radiata* plantation forests. Mean annual sporocarp production of edible species differed significantly among the three stand age groups (F = 7.65; *p* = 0.006; Fig 1A), with the highest mean production levels recorded in old stands (707.18 kg ha^–1^). This value was significantly higher than that of the young stands (*p*-old_*p*-young = 0.0049) but was not significantly different to the yield obtained in medium aged stands (*p*-old_*p*-medium = 0.051). Furthermore, the mean production of fresh sporocarps in medium and young stands did not differ significantly (*p* = 0.460; Fig 1A).

The total mean annual fresh weight production of sporocarps in *P*. *patula* stands was 383.84 kg ha^–1^ year^–1^ and 836.55 kg ha^–1^ year^–1^ in *P*. *radiata* plantations. However, total fresh sporocarp production did not differ significantly between the two plantation types (F = 1.58, *p* = 0.070; Fig 1B).

Among the 24 edible macrofungal species found in study, Saprotrophs were the dominant guild (21 species; 87.5%), whereas only three species were ectomycorrhizal fungi (Table 3). Economically important edible fungal species, such as *Tylopilus niger*, *Suillus luteus*, and *Morchella africana*, were collected (Table 3). The edible species collected according to stand age and stand species, the proportion of the total biomass that each fungal species represented, and their life strategy are presented in Table 3.

**Table 3:** List of edible wild mushrooms and their fresh biomass proportion collected in study plots in *Pinus patula* and *Pinus radiata* plantations located in Menagesha Suba and Wondo Genet, Ethiopia.

Edible macrofungal species	*Pinus patula*			*Pinus radiata*			Guild
	Young	Medium	Old	Young	Medium	Old	
*Agaricus campestroides*	0.0	0.0	3.2	31.2	13.1	0.0	Saprotroph
*Agaricus subedulis*	0.0	0.0	6.8	0.0	0.0	0.0	Saprotroph
*Bovista dermoxantha*	0.0	81.4	0.0	0.0	0.0	0.0	Saprotroph
*Calvatia subtomentosa*	0.0	0.0	8.1	0.0	1.5	2.7	Saprotroph
*Coprinellus domesticus*	33.3	18.6	2.5	0.0	0.0	0.0	Saprotroph
*Hygrophoropsis aurantiaca*	0.0	0.0	8.1	1.0	13.3	9.2	Saprotroph
*Leucoagaricus holosericeus*	0.0	0.0	1.2	0.0	0.0	0.0	Saprotroph
*Leucoagaricus leucothites*	0.0	0.0	8.0	0.0	0.0	0.0	Saprotroph
*Leucoagaricus rubrotinctus*	0.0	0.0	26.1	0.0	0.0	0.0	Saprotroph
*Leucocoprinus cepistipes*	0.0	0.0	1.0	0.0	0.0	0.0	Saprotroph
*Tylopilus niger*	0.0	0.0	35.1	0.0	0.0	0.0	Ectomycorrhiza
*Gymnopilus pampeanus*	0.0	0.0	0.0	6.3	5.3	3.1	Saprotroph
*Lepista sordida*	0.0	0.0	0.0	0.0	4.6	7.8	Ectomycorrhiza
*Lycoperdon cf perlatum*	0.0	0.0	0.0	6.1	4.8	1.9	Saprotroph
*Lycoperdon cf umbrinum*	0.0	0.0	0.0	4.5	10.3	10.5	Saprotroph
*Macrolepiota africana*	0.0	0.0	0.0	6.6	7.0	5.3	Saprotroph
*Morchella africana*	0.0	0.0	0.0	9.3	0.0	8.4	Saprotroph
*Morchella anatolica*	0.0	0.0	0.0	7.9	0.0	11.8	Saprotroph
*Omphalotus illudens*	0.0	0.0	0.0	7.3	8.4	10.1	Saprotroph
*Polyporus badius*	0.0	0.0	0.0	1.0	2.7	1.8	Saprotroph
*Polyporus tenuiculus*	0.0	0.0	0.0	2.7	2.8	4.9	Saprotroph
*Polyporus tuberaster*	0.0	0.0	0.0	7.5	10.0	7.9	Saprotroph
*Schizophyllum commune*	0.0	0.0	0.0	8.6	11.2	4.6	Saprotroph
*Suillus luteus*	0.0	0.0	0.0	0.1	5.0	9.9	Ectomycorrhiza

**Note:** kg ha^-1^year^-1^, Kilogram per hectare per year.

Stand age was significantly correlated with OM in *P*. *patula* (R = 0.98; *p* < 0.05; S1 Fig) and *P*. *radiata* (R = 0.89; *p* < 0.05; S2 Fig) plantations. In *P*. *patula* stands, OM was correlated with both *T*. *niger* (R = 0.92; *p* < 0.05) and total yield (R = 0.97; *p* < 0.05), whereas in *P*. *radiata* stands, OM was correlated with *S*. *luteus* yield (R = 0.8; *p* < 0.05).

### 3.2. Variety of edible mushrooms and edaphic variables

**Fig 2:**
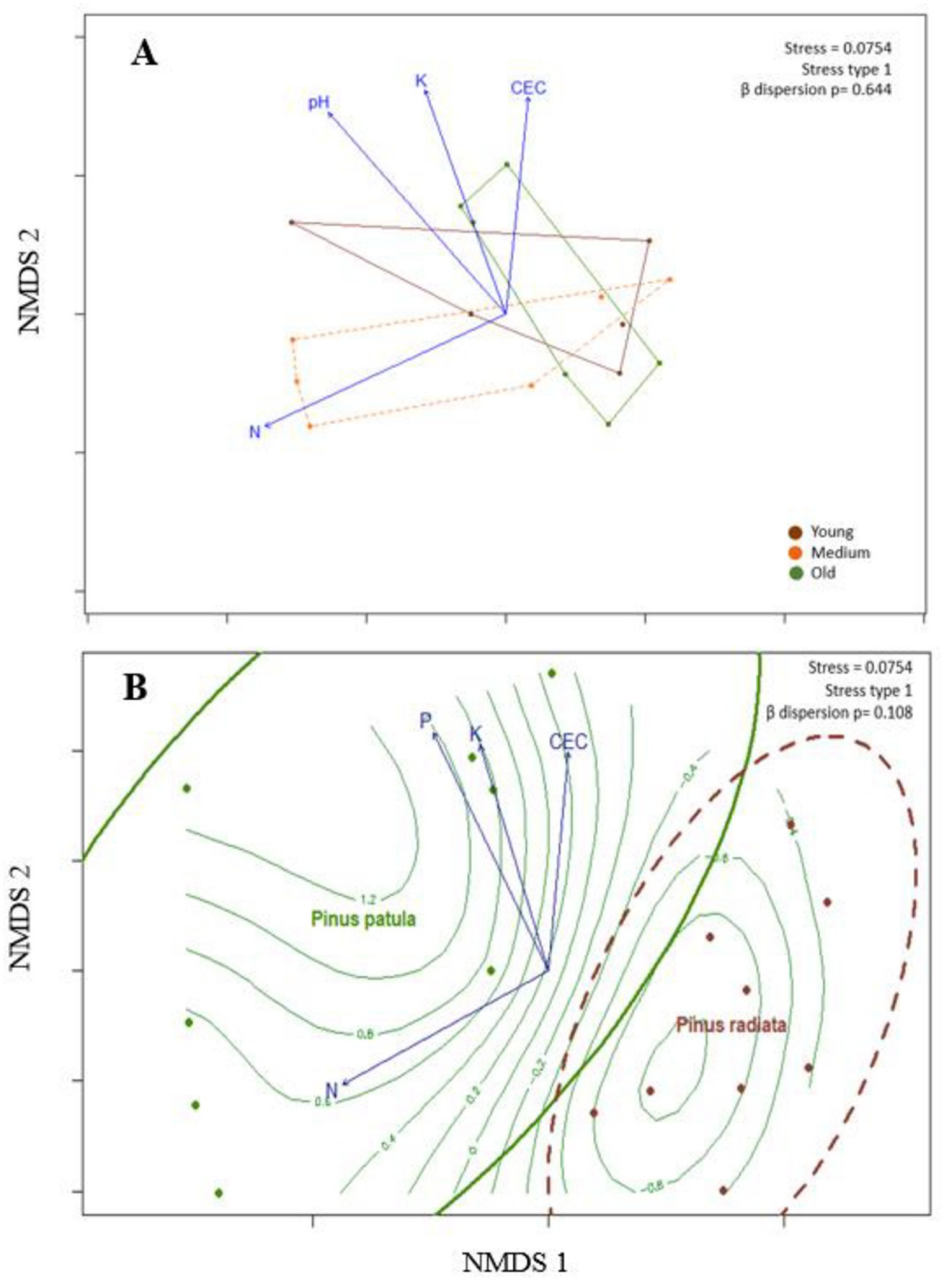
Non-metric Multidimensional Scaling (NMDS) ordination graph with fitted explanatory variables based on dissimilarities calculated using the Euclidean distance of the sporocarp variety in plots based on stand age category (A) and stand type (*Pinus patula* or *Pinus radiata*) (B) in study areas located in Menagesha Suba and Wondo Genet, Ethiopia. **Note:** *Arrows represent environmental variables that were most significantly (p < 0*.*05) related to ordination*. *Soil organic matter content displayed as isolines in B*.

Stand age had a significant influence on the variety of edible sporocarps (F = 1.83, R^2^ = 0.1961, *p* < 0.046; Fig 2A). Explanatory edaphic variables, such as pH, potassium, CEC, and nitrogen, were significantly correlated with sporocarp variety according to stand age (Table 4). NMDS (stress = 0.0754) based on Bray–Curtis distance followed by perMANOVA analyses indicated that the sporocarp variety of *P*. *patula* and *P*. *radiata* plantations differed significantly (F = 7.27, R^2^ = 0.312, *p* = 0.001; Fig 2B). Edaphic variables such as pH, potassium, CEC, nitrogen, and phosphorus were significantly correlated with edible sporocarp variety based on plantation type (*p* < 0.05; Table 4).

**Table 4:** Significance of explanatory variables for sporocarp variety according to plantation type (*Pinus patula* or *Pinus radiata*) and stand age. Edaphic variables with highly significant effects on sporocarp variety are shown in bold (*p* ≤ 0.01).

Variables	Plantation type		Stand age	
	R^2^	*p*-values	R^2^	*p*-values
pH	0.473	**0.008**	0.474	**0.009**
K	0.374	0.030	0.375	0.023
CEC	0.315	0.045	0.315	0.043
N	0.460	0.018	0.461	**0.010**
P	0.490	**0.010**	–	–

Variables: K, potassium; CEC, cation exchange capacity; N, nitrogen; P, phosphorus.

When we analyzed the contribution of individual edible species to the dissimilarity (%) between the different stand age groups of *P*. *patula* and *P*. *radiata* plantations, *Bovista dermoxantha* was the only edible fungal species that contributed to the dissimilarity between young and medium-aged *P*. *patula* stands. *B*. *dermoxantha* also made the biggest contribution to the dissimilarity between young and old *P*. *patula* stands, followed by *T*. *niger* and *Leucoagaricus rubrotinctus* (Table 5). In *P*. *radiata* plantations, *Agaricus campestroides*, *Hygrophoropsis aurantiaca*, and *Schizophyllum commune* made the biggest contribution to the dissimilarity in sporocarp variety between young and medium stands, while *A*. *campestroides*, *Lycoperdon cf umbrinum*, and *S*. *commune* made the biggest contribution to the dissimilarity in sporocarp variety between young and old stands. These analyses suggest that *A*. *campestroides* plays a significant role in young and medium-aged *P*. *radiata* stands, and *B*. *dermoxantha* plays a significant role in medium-aged *P*. *patula* stands.

**Table 5:** Summary of similarity percentage (SIMPER) analyses based on Bray–Curtis measurements showing the cumulative total contribution (75% cut-off) and the contribution (%) of the most influential edible fungal species to the dissimilarity in sporocarp variety between the stand age groups.

Edible fungal species	ICD (%)	CCD (%)
**Young and Medium stands of *Pinus patula***		
*Bovista dermoxantha*	72.3	72.3
**Young and Old stands of *Pinus patula***		
*Tylopilus niger*	29.53	29.53
*Leucoagaricus rubrotinctus*	21.88	51.41
*Coprinellus domesticus*	18.07	69.48
**Medium and Old stands of *Pinus patula***		
*Bovista dermoxantha*	41.38	41.38
*Tylopilus niger*	17.87	59.25
*Leucoagaricus rubrotinctus*	13.24	72.49
*Hygrophoropsis aurantiaca*	10.47	30.96
*Schizophyllum commune*	8.86	39.82
*Morchella africana*	7.74	47.56
*Lycoperdon cf umbrinum*	7.34	54.89
*Polyporus tuberaster*	6.72	61.61
*Morchella anatolica*	6.57	68.18
*Macrolepiota africana*	6.06	74.23
**Young and Old stands of *Pinus radiata***		
*Agaricus campestroides*	24.10	24.10
*Lycoperdon cf umbrinum*	9.28	33.38
*Morchella anatolica*	8.34	41.72
*Suillus luteus*	7.59	49.32
*Polyporus tuberaster*	6.51	55.83
*Hygrophoropsis aurantiaca*	6.39	62.22
*Lepista sordida*	6.02	68.24
*Omphalotus illudens*	5.98	74.22
**Medium and Old stands of *Pinus radiata***		
*Lycoperdon cf umbrinum*	11.62	11.62
*Agaricus campestroides*	10.97	22.59
*Morchella anatolica*	9.83	32.42
*Schizophyllum commune*	8.85	41.28
*Polyporus tuberaster*	8.58	49.86
*Hygrophoropsis aurantiaca*	7.71	57.57
*Morchella africana*	7.03	64.60
*Lepista sordida*	6.03	70.63
**Medium and Old stands of *Pinus radiata***		
*Lycoperdon cf umbrinum*	11.62	11.62
*Agaricus campestroides*	10.97	22.59
*Morchella anatolica*	9.83	32.42
*Schizophyllum commune*	8.85	41.28
*Polyporus tuberaster*	8.58	49.86
*Hygrophoropsis aurantiaca*	7.71	57.57
*Morchella africana*	7.03	64.60
*Lepista sordida*	6.03	70.63
**Host: *Pinus patula*** Vs ***Pinus radiata***		
*Bovista dermoxantha*	13.89	13.89
*Coprinellus domesticus*	9.30	23.19
*Agaricus campestroides*	9.20	32.39
*Tylopilus niger*	5.99	38.39
*Omphalotus illudens*	5.38	43.76
*Polyporus tuberaster*	5.31	49.07
*Polyporus tuberaster*	5.29	54.36
*Lycoperdon cf umbrinum*	5.10	59.46
*Schizophyllum commune*	4.73	64.19
*Hygrophoropsis aurantiaca*	4.44	68.64
*Leucoagaricus rubrotinctus*	4.10	72.74

**Note:** ICD, individual contribution to the dissimilarity (%); CCD, cumulative contribution to the dissimilarity (%).

We found that *T*. *niger*, *B*. *dermoxantha*, *Agaricus subedulis*, and *Coprinellus domesticus* were specific to *P*. *patula* stands, while *Suillus luteus*, *Morchella africana*, and *Macrolepiota africana* were found exclusively in *P*. *radiata* stands. However, *A*. *campestroides*, *Calvatia subtomentosa*, and *Hygrophoropsis aurantiaca* were found in both *P*. *patula* and *P*. *radiata* plantations (Table 3).

**Fig 3:**
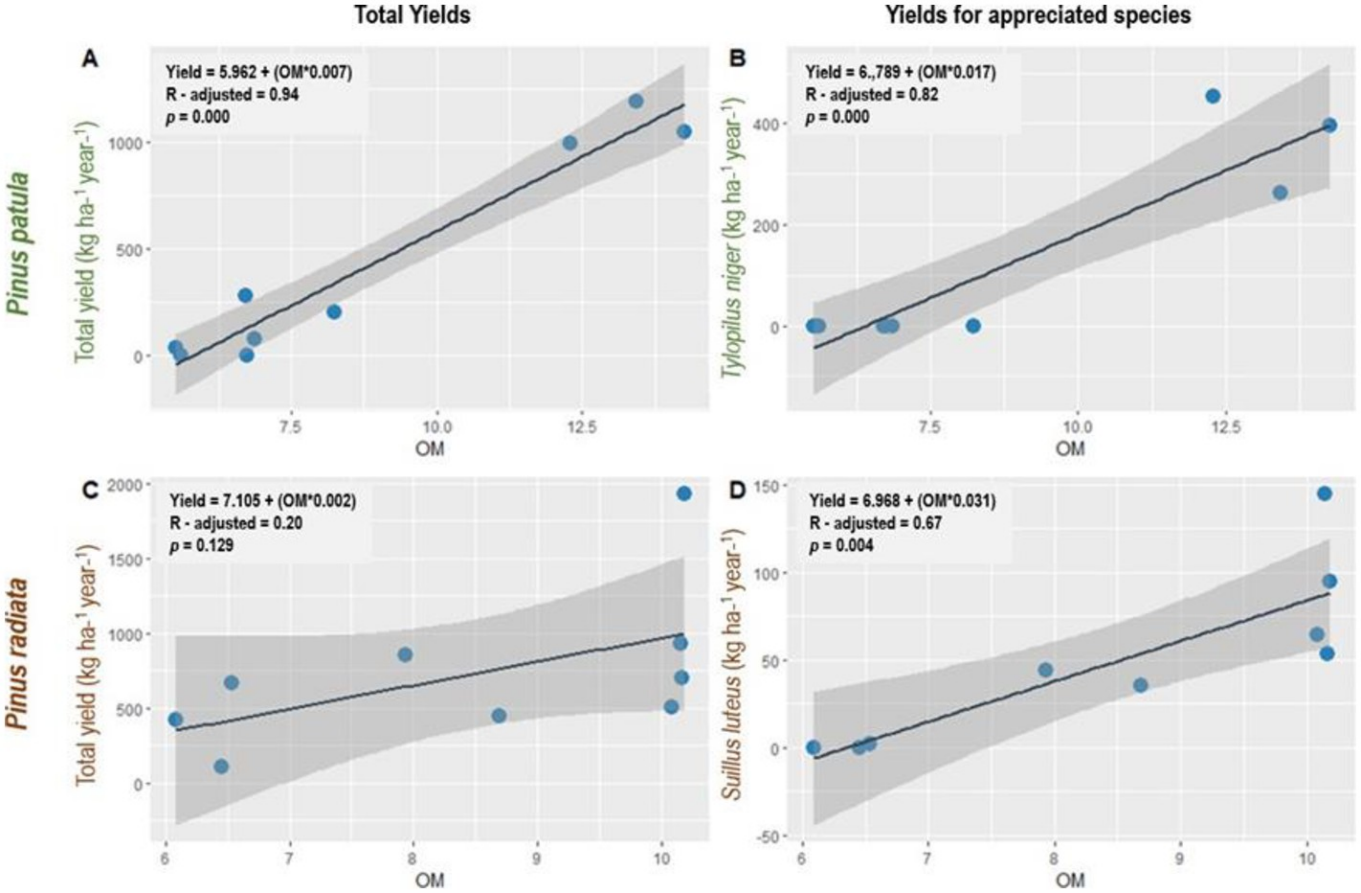
Linear regression models of observed and predicted total edible sporocarp yield values in Pinus patula (A) and Pinus radiata (C) plantations and total sporocarp yield values of highly appreciated macrofungal species (B and D) in study areas located in Menagesha Suba and Wondo Genet, Ethiopia. **Note:** *Blue circles represent observed soil organic matter (OM) values*, *black lines indicate line fit plots*, *and shaded areas indicate 95% confidence intervals*.

Linear regression models indicated that total sporocarp yields in *P*. *patula* and *P*. *radiata* forests and of specific valuable fungal species were significantly predicted by OM (*p* < 0.05; Fig 3). In both plantation types, the model fits the data well and OM explained 94% and 20% of the variation in total sporocarp production in *P*. *patula* and *P*. *radiata* stands, respectively. Similarly, OM explained 82% and 67% of the variation in *T*. *niger* and *S*. *luteus* sporocarp production, respectively. The sign of the coefficient was positive for both, which indicates that as OM increases, sporocarp production also increased. Thus, mean sporocarp production would increase for every one unit increase in OM in the studied plantations.

## 4. Discussion

The demand for mushrooms has been steadily increasing over the past decade [3]. This trend highlights the commercial value of forests, which can be enhanced through managed timber harvesting that could potentially enhance the habitat for the production of valuable edible mushrooms [74]. Although previous studies have reported the availability of wild fungi in Ethiopia, information about the type and potential yields of wild edible fungal species in exotic plantations is scarce. The aim of this study was to analyze how the age of short-rotation *Pinus* plantations affects the production of edible mushrooms. We collected a total of 24 edible fungal species. Most of these edible species (21) were saprotrophs. Some species were collected exclusively from *P*. *patula* (e.g., *T*. *niger*) or *P*. *radiata* (e.g., *S*. *luteus*) stands. This supports previous findings by [2] that some valuable fungal species are found exclusively in a particular forest system, indicating that different fungal species have unique ecological requirements for fructification or sporocarp production [75]. Thus, understanding the ecological preferences of various fungal species is essential for designing effective management strategies to promote their growth and conservation in different plantation systems.

As expected, mushroom yields were significantly influenced by stand age, which supports the findings of previous studies of fungal communities in Ethiopian forests [18,76]. Total sporocarp fresh weight yields were significantly higher in old stands than those in young stands, indicating that sporocarp yields increased as *Pinus* stands of both plantation types matured. This can be explained by changes in the physicochemical characteristics of the soil as the stands develops. For example, as a forest stand matures, the humus layer develops [77–80] and the forest soil increases its capacity to buffer temperature and moisture [78]. Such conditions could enhance fungal growth and fruiting, particularly of saprotrophic fungi [81], which dominated the fungal communities in our collections. In addition, increased humidity and OM accumulation along the age gradient of *P*. *patula* and *P*. *radiata* stands may lead to a more diverse spatial distribution, which could enhance fruiting, particularly in older stands [82,83]. Furthermore, stand age is one of the most important factors influencing fungal community variety in plantation forests [45,84]. An increase in the soil OM content over time enhances soil microbial activities, and the variety of microhabitats increases as the canopy closes, which in turn increases sporocarp production [85]. This notion is consistent with the findings of [83], who reported that well-developed stands with higher levels of canopy cover had greater levels of fungal diversity.

Stand age also significantly affected the most valuable edible species. Approximately 33% of the species collected from all plantations have economic significance and are marketable [3], including *Agaricus* sp., *Morchella* sp., *Suillus* sp., *Lepista* sp., and *Tylopilus* sp. [3]. Most of these species were found in middle-aged and old stands, which agrees with previous studies indicating that older stands harbor valuable species and have higher sporocarp yields than younger stands [39]. Previous reports have also recognized these edible fungal species as commercial NTFPs [86], which could potentially enhance the economic performance of forests [87] and encourage local communities to plant and manage more plantations in their surroundings. Furthermore, in some tropical countries, some of these fungal species are exported as forest products to generate income [53]. Despite the comparatively low total sporocarp yields obtained from the *Pinus* plantations assessed in this study [39], the sporocarp biomass obtained provides an insight into the potential production levels of the two plantation types, particularly of the marketable species. Thus, forest managers in Menagesha Suba and Wondo Genet should consider the balance between timber production and fungal conservation for environmental and production purposes. Consequently, maintaining *Pinus* trees as shelter instead of clear-cutting the plantation may be a more effective way of preserving and promoting the diversity and production of edible fungi. Thus, stand age had a significant impact on the total yield and on the yield of the most valuable species. Furthermore, stand age is one of the most important factors influencing fungal community variety in plantation forests [45,84]. Studies have shown that fungal diversity tends to increase with stand age, with older forests having a more diverse and complex fungal community compared with younger forests [39,88].

*B*. *dermoxantha* and *A*. *campestroides* may play important ecological roles in either young or old *Pinus* plantations. As saprophytes, *B*. *dermoxantha* and *A*. *campestroides* decompose dead plant matter, releasing nutrients that can be taken up by growing trees [89]. This process helps to build the nutrient-rich soils that trees need to grow. Saprotrophs play important roles in nutrient cycling and in the maintenance of healthy soils. As trees age and begin to decline, fungi help to break down dead plant material, releasing trapped nutrients back into the soil [90]. This cycle is important in maintaining the long-term health and sustainability of *Pinus* plantations.

In general, most of the collected edible macrofungal species made a large contribution to dissimilarities in the edible fungal community between young and old stands of *Pinus*. This suggestion is in line with findings that revealed that as forests age, the structure and variety of the vegetation changes, which can affect fungal community variety and diversity [91,92]. Furthermore, the tree species planted in the plantation can also influence fungal community variety [93].

Our analyses also revealed that soil conditions, referred to as edaphic variables, played a significant role in shaping the sporocarp variety in the studied stands [94,95]. This is because different fungal taxa are likely to respond to edaphic variables in different ways, depending on their characteristics [96,97], and, thus, in turn, the variety of fungal communities is directly correlated with soil parameters [98]. Among the edaphic variables, pH was significantly associated with the total sporocarp composition. We also observed that some edaphic variables were associated with the higher end of the pH isolines, indicating that nitrogen, phosphorus, potassium, and CEC are directly related to pH gradients [99]. This might be because soil pH is an important factor that governs the variety of sporocarps in forests because it affects the availability of other essential nutrients such as nitrogen, phosphorus, and potassium [6,100]. The solubility and availability of these nutrients are also influenced by soil pH values [101,102]. Thus, changes in pH can directly impact them, thus changing the variety of fungal communities in the forest ecosystem [103–105]. Nitrogen and phosphorus availability in the soil can also affect the distribution and abundance of fungal species in the forest [106]. Some fungi are better adapted to low-nitrogen environments and can thrive in nutrient-poor soils [107]. Potassium is another important nutrient for macrofungal species because it regulates osmotic pressure, maintains cell turgor, and balances cellular pH [108]. Thus, the availability of nitrogen, phosphorus, and potassium in the soil can influence the growth and development of fungal hyphae and affect the ability of macrofungi to compete with other microorganisms for soil resources [109,110]. Our study also revealed that the CEC is a contributing factor to sporocarp production. Although the specific role that CEC plays in sporocarp production is not yet fully understood, [111] noted that fungal species richness tends to be low when CEC is high. In our study, the majority of sporocarps collected in *P*. *radiata* stands were associated with low CEC values. This could be because soils with a high CEC are less likely to release base cations [112], which is an important factor in the distribution of macrofungal species. Base elements are crucial in many physicochemical processes, such as photosynthesis and, therefore, can impact plant photosynthesis and the amount of carbon available to fungi in the soil [113,114].

As anticipated, distinct communities of edible sporocarp species were found in *P*. *patula* and *P*. *radiata* plantations. Differences in community variety can be attributed to variations in environmental conditions between *P*. *patula* and *P*. *radiata* plantations, such as pH, nutrient availability, and moisture, which can impact the growth and survival of different fungal species. For instance, certain fungal species may thrive in acidic soils while others may prefer more alkaline conditions [115,116]. In our study, the heterogeneous soil environment, coupled with high levels of rainfall, could create microhabitats in which specific fungal species would be able to grow and survive in a particular plantation type [2]. For instance, we found that *T*. *niger*, *B*. *dermoxantha*, *A*. *subedulis*, and *Coprinellus domesticus* were specific to *P*. *patula* stands, while *S*. *luteus*, *Morchella africana*, and *Macrolepiota africana* were exclusive to *P*. *radiata* stands.

Three of the 24 edible fungal species collected in this study were ectomycorrhizal, (i.e., *T*. *niger*, *L*. *sordida*, and *S*. *luteus*), and could form mutualistic relationships with the host tree species. These mutualistic species are important for maintaining the health and productivity of plantation forests in our study areas. *Agaricus campestroides*, *Calvatia subtomentosa*, and *Hygrophoropsis aurantiaca* were common to both *P*. *patula* and *P*. *radiata* plantations, indicating that these genera might be characterized as generalists. Generalist fungal species are important for maintaining ecosystem functioning, biodiversity conservation, and can act as indicator species in forest ecosystems [117,118]. Thus, the presence of generalist fungal species can provide valuable information about the health and condition of forest ecosystems and indicate the role of plantation tree species in supporting a range of fungal species, which might be crucial for maintaining the overall functioning of forest ecosystems [43,119,120].

In addition, we found a correlation between OM and *T*. *niger* and *S*. *luteus* sporocarp production in *P*. *patula* and *P*. *radiata* plantations, respectively. This relationship could be attributed to the fact that mycorrhizal fungi tend to expand their mycelial network at the soil interface [121,122], where OM can influence the growth and network formation of mycelia [123]. Although a single growing season’s worth of data is insufficient to establish a clear relationship between soil OM and macrofungi, our preliminary findings suggest that soil OM content is directly associated with the overall richness and abundance of macrofungal species, a finding that is consistent with previous studies conducted by [39,124].

## 5. Conclusions

In this study, we investigated the influence of stand age on edible sporocarp production in Ethiopian short-rotation *Pinus* plantations. Stand age was shown to make a significant contribution to variations in fungal sporocarp production. Most of the collected species were found in old stands of *P*. *patula* and *P*. *radiata*, likely due to the greater availability of suitable substrates for fungal growth and sporocarp production in older stands than in younger stands. Therefore, preserving mature *Pinus* stands as shelter rather than clear-cutting them may be a more effective approach to promoting valuable fungal species and their sporocarp production. Moreover, edaphic variables were also found to be significantly correlated with sporocarp production in both *P*. *patula* and *P*. *radiata* plantations, suggesting that nutrient availability in the soil can influence edible fungal variety and sporocarp production. Thus, understanding the nutrient requirements of different fungal species can help us to better manage and conserve forest plantations to produce both timber and mushrooms as NTFPs. Hence, using a tree retention management approach in short-rotation *Pinus* plantations could be beneficial, leaving patches of live standing mature trees after the final rotation cut, which could increase habitat availability for saprophytic fungi owing to increased soil fertility, thereby increasing fungal diversity and edible sporocarp production. This method also provides live standing trees for mycorrhizal species such as *T*. *niger* and *S*. *luteus* after the final rotation cut, resulting in greater mycorrhizal sporocarp production. Some of the species collected in *P*. *patula* and *P*. *radiata* plantations also have potential economic value as the collections of edible mushrooms could create livelihoods in areas where economic alternatives may be limited. Moreover, the sale of mushrooms can generate revenue, and, hence, could provide additional income to forest managers and benefit especially small-scale collectors from the local communities. Furthermore, mushrooms also play a vital role in fostering sustainable forest management by enhancing soil health, nutrient cycling, and acting as indicators of ecosystem well-being. These contributions underscore the holistic significance of mushrooms in maintaining the vitality and economic sustainability of plantation forests. Thus, the approach of preserving mature *Pinus* stands could provide additional income opportunities for the rural population through the sale of mushrooms while promoting biodiversity conservation by balancing timber and fungal production.

## Supporting information

S1 Fig Scatter plot matrices showing Pearson correlation coefficients between edaphic variables and significance levels for total sporocarp production and edaphic parameters in the Pinus patula stands. Abbreviations: EC, Electrical Conductivity; K, Potassium; Ca, Calcium; Mg, Magnesium; CEC, Cation Exchange Capacity; OM, Organic Matter; N, Nitrogen; P, Phosphors. On the bottom of the diagonal, bi-variate scatter plots with a fitted line are displayed. On the top of the diagonal, the value of the Pearson correlation is shown, plus the significance level of the p-values, which are indicated by asterisks. p-values: ***, < 0.001; **, < 0.01; and *, < 0.05.
(TIF)

S2 Fig Scatter plot matrices showing Pearson correlation coefficients between edaphic variables and significance levels for total sporocarp production and edaphic parameters in the Pinus radiata stands. Abbreviations: EC, Electrical Conductivity; K, Potassium; Ca, Calcium; Mg, Magnesium; CEC, Cation Exchange Capacity; OM, Organic Matter; N, Nitrogen; P, Phosphors. On the bottom of the diagonal, bi-variate scatter plots with a fitted line are displayed. On the top of the diagonal, the value of the Pearson correlation is shown, plus the significance level of the p-values, which are indicated by asterisks. p-values: ***, < 0.001; **, < 0.01; and *, < 0.05.
(TIF)

S1 File Edible wild mushrooms and their total fresh biomass (kg ha^-1^year^-1^) collected in study plots in *Pinus patula* and *Pinus radiata* plantations located in Menagesha Suba and Wondo Genet, Ethiopia. (XLSX)
