# Supplementary figures and images for: Green trees preservation: A sustainable source of valuable mushrooms for Ethiopian local communities
Source: PLoS One. 2023 Nov 29;18(11):e0294633. doi: 10.1371/journal.pone.0294633 (PMC10686473; doi:10.1371/journal.pone.0294633)

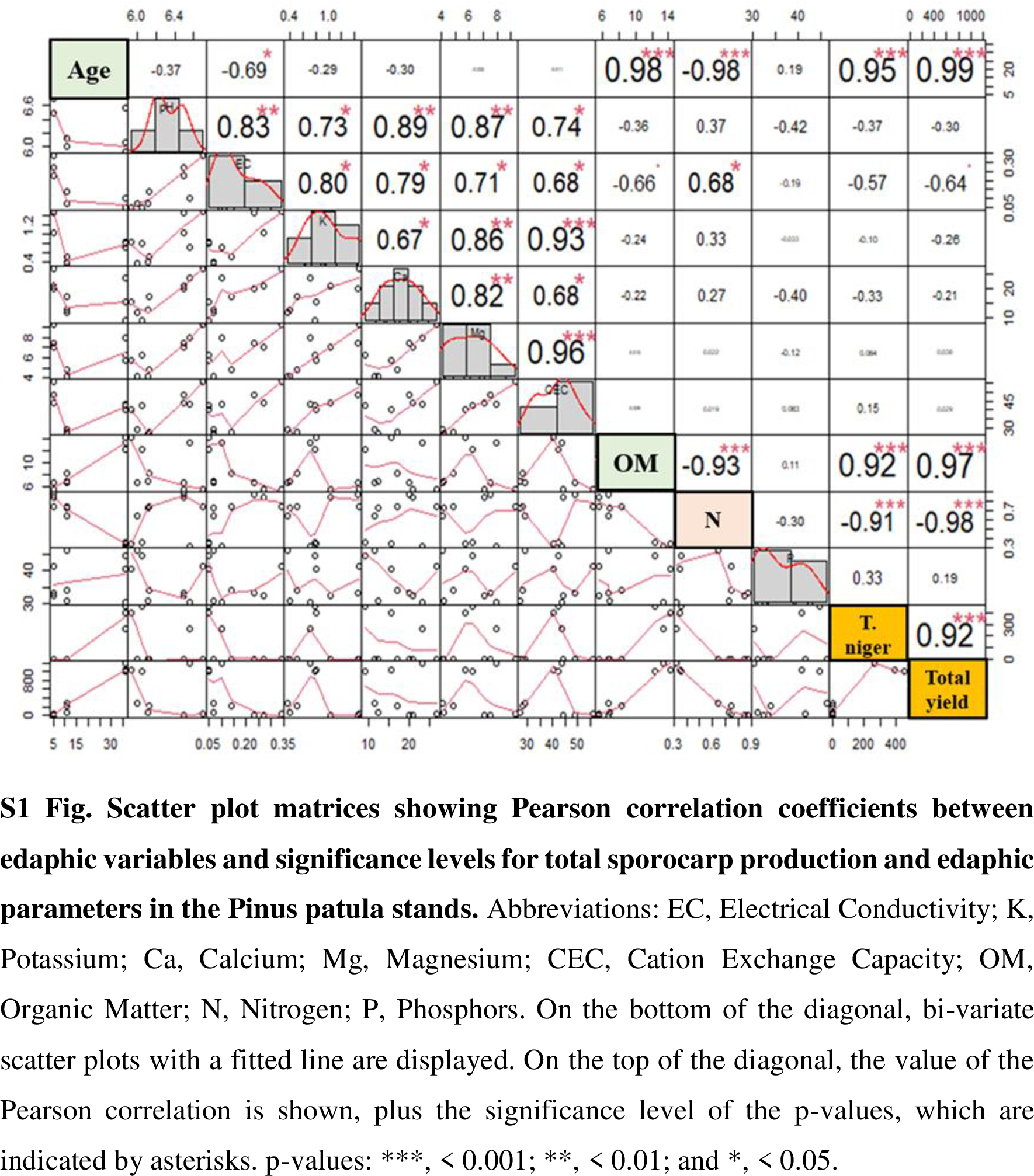

Supplement: S1 Fig — Abbreviations: EC, Electrical Conductivity; K, Potassium; Ca, Calcium; Mg, Magnesium; CEC, Cation Exchange Capacity; OM, Organic Matter; N, Nitrogen; P, Phosphors. On the bottom of the diagonal, bi-variate scatter plots with a fitted line are displayed. On the top of the diagonal, the value of the Pearson correlation is shown, plus the significance level of the p-values, which are indicated by asterisks. p-values: ***, < 0.001; **, < 0.01; and *, < 0.05. (TIF) [file pone.0294633.s001.tif]

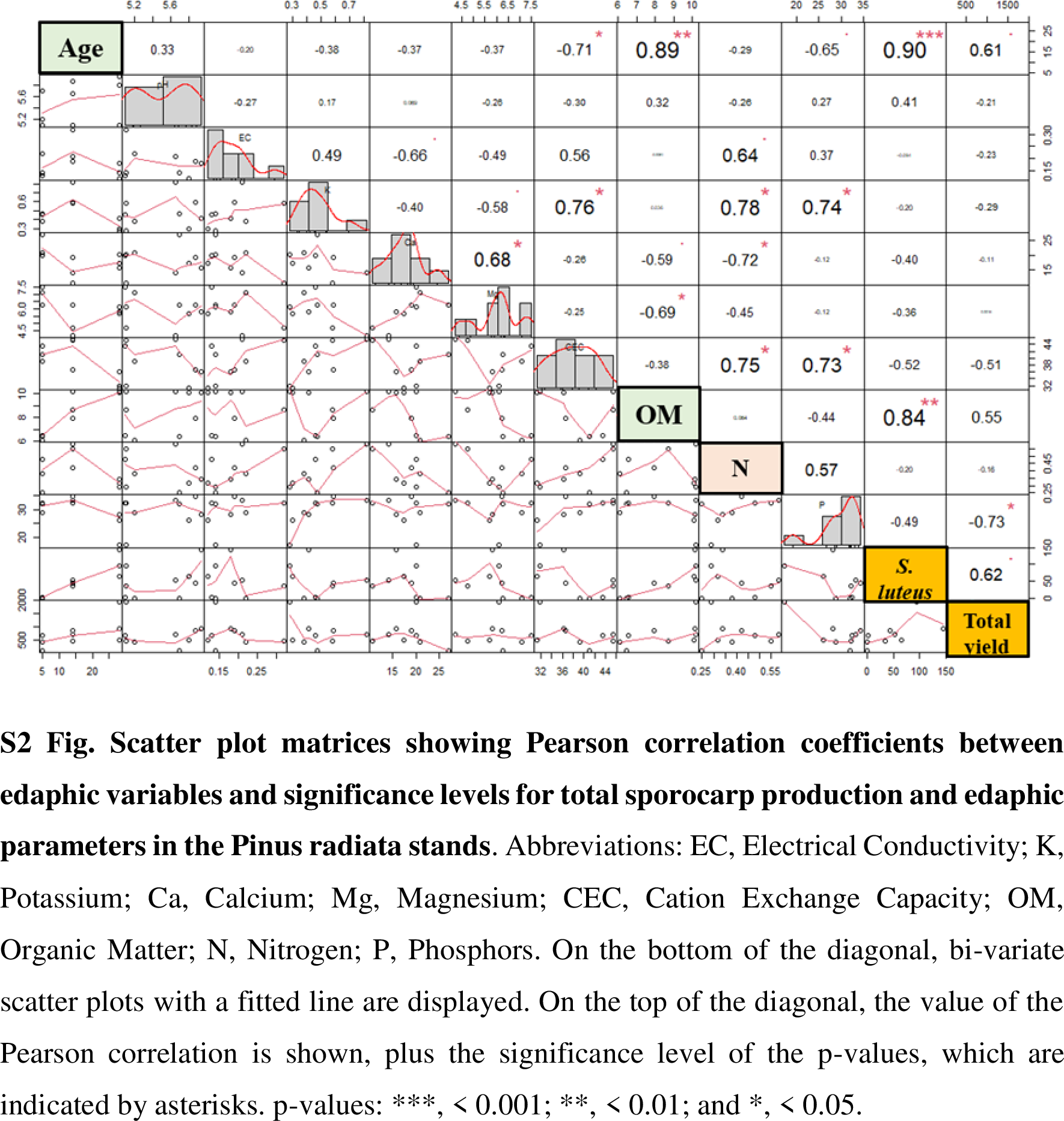

Supplement: S2 Fig — Abbreviations: EC, Electrical Conductivity; K, Potassium; Ca, Calcium; Mg, Magnesium; CEC, Cation Exchange Capacity; OM, Organic Matter; N, Nitrogen; P, Phosphors. On the bottom of the diagonal, bi-variate scatter plots with a fitted line are displayed. On the top of the diagonal, the value of the Pearson correlation is shown, plus the significance level of the p-values, which are indicated by asterisks. p-values: ***, < 0.001; **, < 0.01; and *, < 0.05. (TIF) [file pone.0294633.s002.tif]
